# Supplementary figures and images for: Milan’s forgotten epidemic of summer 1629, a few months before the last great plague: An investigation into the possible cause
Source: PLoS One. 2023 Jun 8;18(6):e0279218. doi: 10.1371/journal.pone.0279218 (PMC10249863; doi:10.1371/journal.pone.0279218)

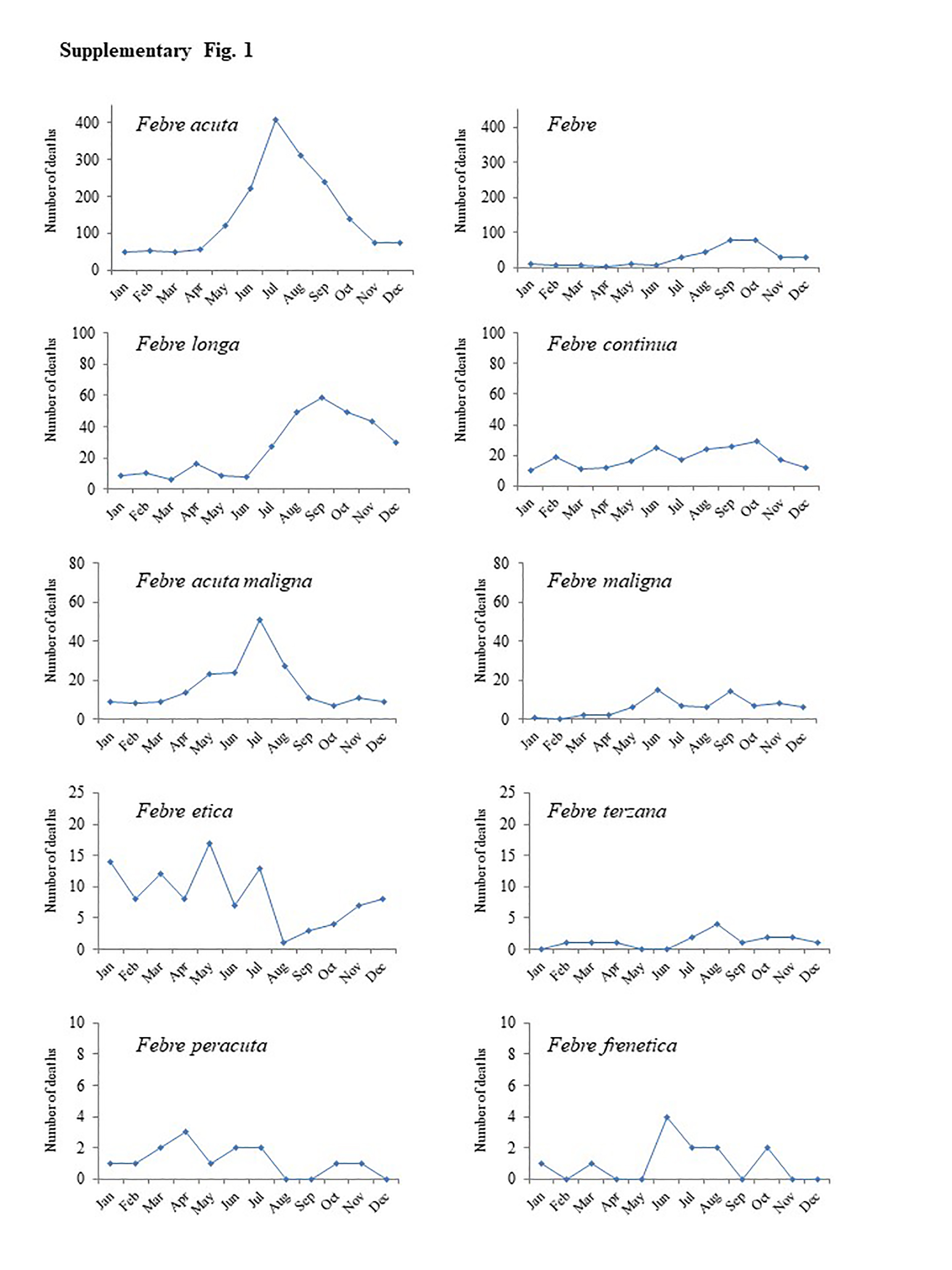

Supplement: S1 Fig — (JPG) [file pone.0279218.s002.jpg]
